# Space closure rate and anchorage control during maxillary anterior en-masse retraction using bidimensional versus conventional sliding mechanics

**DOI:** 10.6026/973206300222069

**Published:** 2026-04-30

**Authors:** K Muhammadali, Arvind Nair, Virendra Vadher, Shalabh Baxi, Shweta Singh, Chhaya Barapatre

**Affiliations:** 1 Department of Orthodontics and Dentofacial Orthopedics, Government Dental College, Raipur, Chhattisgarh, India

**Keywords:** Orthodontic space closure, maxillary anterior retraction, bidimensional orthodontic technique, sliding mechanics, torque expression

## Abstract

Efficient orthodontic space closure depends on optimal control of friction, anchorage and anterior torque. Therefore, it is of interest to compare four maxillary anterior retraction techniques-conventional sliding mechanics, bidimensional wire technique, bidimensional slot technique and a double-slot bracket system-by evaluating the rate and amount of space closure, anchorage loss, molar rotation and torque control. Forty patients were equally divided into four groups and treatment effects were assessed using standardized model analysis and cephalometric measurements. Bidimensional and double-slot techniques demonstrated significantly faster rates of space closure and superior torque control compared with conventional sliding mechanics. At the same time, anchorage loss and molar rotation were comparable among all groups. Thus, we show the biomechanical efficiency of bidimensional retraction techniques with conventional mechanics during maxillary anterior space closure.

## Background:

Space closure is a critical phase of orthodontic treatment, particularly in extraction cases where efficient anterior retraction must be balanced with anchorage preservation and torque control. Contemporary orthodontics primarily employs sliding (frictional) mechanics or frictionless loop mechanics for space closure [1]. Following the introduction of pre-adjusted edgewise appliances, sliding mechanics gained widespread acceptance due to reduced need for complex wire bending and improved procedural efficiency [21]. However, sliding mechanics is influenced by resistance to sliding at the bracket- archwire interface. This resistance comprises friction, binding and notching and is affected by bracket slot dimensions, archwire size and alloy, ligation method and inter-bracket distance [3, 4]. Experimental studies demonstrate that increased wire-slot engagement significantly elevates resistance, thereby decreasing the biologically effective force available for tooth movement [5]. Nevertheless, Southard *et al.* emphasized that friction itself does not inherently increase anchorage loading when teeth are free to slide; rather, anchorage loss depends on the overall force system applied [6]. Anterior retraction may be performed using either a two- step approach (canine retraction followed by incisor retraction) or en masse retraction of the entire anterior segment. A systematic review by Rizk *et al.*reported no clinically significant difference in anchorage loss between these approaches when similar biomechanics are used [7]. Randomized clinical trials have corroborated these findings, demonstrating comparable anchorage outcomes between techniques [8, 9]. More recent trials comparing frictional and frictionless mechanics also indicate that, despite biomechanical differences, anchorage loss remains within a similar clinical range under conventional anchorage conditions [10, 11 and 12]. Anchorage, defined as resistance to unwanted tooth movement, remains fundamental in extraction therapy. Without skeletal anchorage, maxillary molar mesialization of approximately 1-3 mm is commonly reported during space closure [13]. Biomechanical principles established by Burstone and Koenig highlight the importance of maintaining an optimal moment-to-force (M/F) ratio to prevent posterior tipping and anchorage loss [14]. Finite element analyses further confirm that reducing friction alone does not eliminate anchorage demands, as posterior teeth continue to experience reciprocal forces during anterior retraction [15, 16- 17]. A significant limitation of sliding mechanics is loss of anterior torque, primarily due to torsional play between the archwire and bracket slot. Dimensional variability in 0.022-inch systems may produce torsional discrepancies exceeding 10°C, substantially reducing effective torque expression [18]. Experimental investigations have shown that torque efficiency is influenced by slot size, archwire geometry and material properties, with smaller slots and rectangular wires generating higher torque moments [19, 20 and 21]. Finite element simulations further demonstrate that increased slot-wire clearance may require longer power arms to achieve controlled bodily anterior movement [22]. To address both frictional resistance and torque limitations, bidimensional concepts were developed. The original bimetric system combined smaller anterior brackets with larger posterior slots to improve anterior control while facilitating posterior sliding [23]. Gianelly *et al.*later formalized the bidimensional edgewise technique, selectively engaging the anterior segment while allowing posterior clearance to reduce resistance [24]. Clinical and typodont studies indicate that bidimensional systems may enhance preservation of anterior torque during extraction space closure compared with conventional mechanics [25]. Therefore, it is of interest to compare these four maxillary anterior retraction modalities in terms of rate of space closure, anchorage loss, molar rotation and torque control.

## Materials and Methods:

This prospective clinical study was conducted in the Department of Orthodontics and Dentofacial Orthopaedics at the Government Dental College, Raipur. Patients reporting to the outpatient department who fulfilled the eligibility criteria were recruited after obtaining written informed consent. The study protocol was approved by the Institutional Ethics Committee (Approval No. ECR/07/GDC/CG/2023). The study duration was approximately 24 months following ethical clearance. Forty patients aged 15-25 years requiring bilateral maxillary first premolar extraction were included. Eligible participants presented with Class I or Class II malocclusion, minimal or no maxillary crowding and no requirement for orthognathic surgery. Patients with skeletal malocclusion, a history of jaw or dental trauma, periodontal disease, medically compromised conditions or prolonged medications affecting bone metabolism were excluded. After leveling and alignment, subjects were randomly assigned to four groups of ten patients each. Group 1 received the bidimensional wire technique with 0.022-inch MBT brackets and a 0.018 x 0.022-inch stainless steel archwire incorporating a 90°C twist distal to the canine. Group 2 used the bidimensional slot technique with 0.018-inch anterior and 0.022-inch posterior brackets, with a 0.018 x 0.025-inch stainless steel archwire. Group 3 employed double-slot brackets that engaged the 0.018-inch slot anteriorly and the 0.022-inch slot posteriorly, with a 0.018 x 0.025-inch stainless steel archwire. Group 4 used conventional sliding mechanics with a 0.019 x 0.025-inch stainless steel archwire in 0.022-inch MBT brackets. Anchorage reinforcement was achieved in all groups using bonded second molars and a transpalatal arch. En masse anterior retraction was performed with nickel-titanium closed-coil springs delivering 150 g per side. Patients were reviewed every 4 weeks to verify force levels. Records, including lateral cephalograms and maxillary study models, were obtained before (T0) and after (T1) space closure. Space closure was measured on study models using calipers to determine the linear distance between the extraction sites over time and the rate of closure was calculated by dividing the space closed by the duration of retraction. Anchorage loss was assessed by measuring mesial movement of the maxillary first molars on models and cephalograms. Molar rotation was evaluated using the angulation of the molar relative to the S-N and Ba-N plane. Incisor torque was analyzed cephalometrically by measuring the inclination of maxillary central incisor bracket base relative to the arch wire and assessing angular changes from T0 to T1.

## Statistical analysis:

Data obtained from cephalometric analysis and study models were statistically analyzed using IBM SPSS Statistics version 25. Normality of distribution was checked using the Shapiro-Wilk test. As the data were not normally distributed, non-parametric tests were employed. The Kruskal-Wallis test was used to compare intergroup differences across all outcome variables, including the amount and rate of space closure, anchorage loss, molar rotation and changes in anterior torque. The level of statistical significance was set at p < 0.05.

## Results:

A total of 40 patients were included in the study, equally distributed among the four groups. Model and cephalometric analyses were performed to evaluate the amount and rate of space closure, anchorage loss, molar rotation and changes in anterior torque. Intergroup comparisons using the Kruskal-Wallis test are presented in Table 1 (see PDF). For model analysis, the rate of space closure differed significantly among the groups (H = 12.274, p = 0.006), with the bidimensional wire, bidimensional slot and double-slot groups exhibiting faster closure compared with conventional mechanics. Other model parameters, including canine-second premolar distance, first molar-third rugae distance and molar rotation (mesial and distal surfaces), showed no statistically significant differences between groups. Cephalometric evaluation indicated that changes in anterior torque differed significantly among the groups (H = 8.831, p = 0.032). In contrast, other variables, including canine-second premolar distance, rate of space closure, first molar-pterygoid vertical distance and molar rotation (SN plane and Ba-N plane), were not significantly different. Post-hoc pairwise comparisons using the Mann-Whitney U test with Bonferroni correction were conducted for the significant parameters (Table 2 - see PDF). For the rate of space closure, the bidimensional wire, bidimensional slot and double-slot groups each showed significantly faster space closure compared with the conventional retraction group. Similarly, for torque changes, the bidimensional wire group demonstrated significantly better torque preservation than conventional mechanics. In contrast, differences between the bidimensional slot and double-slot groups and conventional mechanics did not reach the Bonferroni-adjusted significance level. These results indicate that bidimensional wire and related friction-reducing techniques enhance the rate of anterior retraction and torque control compared with conventional sliding mechanics. At the same time, anchorage and molar rotation remain largely unaffected by the choice of retraction system.

## Discussion:

Efficient orthodontic space closure requires optimal force delivery, preservation of anchorage and effective torque expression. Conventional sliding mechanics typically employs full-size rectangular stainless-steel wires to achieve three-dimensional control; however, increased wire-slot engagement elevates resistance to sliding and reduces biomechanical efficiency [3, 5]. Laboratory investigations consistently show that frictional resistance increases with greater engagement and with the use of rectangular wire [26]. In the present study, model analysis demonstrated significantly higher rates of space closure in the bidimensional and double-slot groups compared with the conventional group. These findings are biomechanically consistent with Gianelly *et al.*original clinical observations that selective posterior disengagement reduces frictional resistance and enhances retraction efficiency [24]. Kusy further emphasized that increased wire-slot engagement can reduce effective force delivery by up to 25-60%, reinforcing the role of appliance design in retraction efficiency [27]. Similarly, Gnaneswar and Sridhar reported significantly greater rates of space closure with dual-dimensional wires compared with conventional rectangular wires during mini-implant-supported retraction, attributing the improvement to reduced resistance to sliding and more efficient force transmission [28]. Randomized clinical evidence indicates that, when biologically acceptable force levels are applied, the total amount of space closure achieved is largely independent of appliance design [29]. Similarly, recent trials comparing frictional and frictionless mechanics reported differences in biomechanics but comparable overall treatment outcomes [10, 11]. This explains why the present study observed comparable overall space closure among groups despite differences in retraction rate. Anchorage loss did not differ significantly among groups, although the conventional group showed slightly higher mean values. This aligns with systematic reviews demonstrating that conventional anchorage methods, including transpalatal arches, provide limited resistance against molar mesialization [13, 30]. Finite element analyses by Kojima and Fukui support the observation that reducing friction does not eliminate anchorage loss because posterior teeth experience reciprocal mesial forces regardless of sliding resistance [15, 16]. Therefore, anchorage preservation depends more on force system optimization and reinforcement strategies than on bracket design alone. Molar rotation remained minimal and statistically insignificant among groups. Randomized clinical studies evaluating canine retraction mechanics have similarly reported limited molar rotational change during space closure [31, 32]. These findings indicate that molar rotation is more closely related to force vector direction and activation protocol than to bracket slot configuration. Torque control represented the most distinct difference among techniques. The bidimensional wire group exhibited superior torque preservation compared with conventional mechanics. This is consistent with dimensional analyses showing substantial torsional play in conventional 0.022-inch systems [18]. Arreghini *et al.*demonstrated that torque expression is significantly limited by slot oversizing and archwire under sizing, independent of wire material [19]. Papageorgiou *et al.*further confirmed that 0.018- inch systems generate significantly higher torque moments than 0.022-inch systems due to reduced play [20]. Finite element modeling by Tominaga *et al.*highlighted that larger slot-wire discrepancies require longer power arms to compensate for torque inefficiency [22]. Li *et al.*, Shayeb *et al.* and Wichelhaus *et al.*reported significantly improved preservation of maxillary incisor torque with bidimensional-wire and bidimensional-slot techniques compared with conventional sliding mechanics, highlighting the advantage of selective anterior engagement with posterior clearance in enhancing torque efficiency while reducing resistance to sliding [25, 33 and 34]. Although conventional sliding mechanics remain clinically effective, they may result in slower retraction and greater loss of anterior torque in high-demand cases. Limitations include small sample size, two-dimensional cephalometric assessment, biological variability, lack of skeletal anchorage and absence of long-term stability evaluation. Future studies using larger samples and three-dimensional imaging are warranted.

## Conclusion:

All four retraction techniques achieved clinically acceptable space closure. Bidimensional and double-slot systems demonstrated faster retraction and superior torque control without increasing anchorage loss or molar rotation. The bidimensional wire technique showed the best overall biomechanical performance. Thus, appliance selection should be tailored to clinical objectives and operator expertise.

## Declarations:

## Author contribution:

All authors made substantial contributions to this study. Muhammadali K conceived the research concept, developed the technique and led data collection. Virendra Vadher supervised patient examination and selection, evaluated the methodology and performed the statistical analysis. Shalabh Baxi conducted the literature review, interpreted clinical records and assisted in manuscript preparation. Muhammadali K and Shweta Singh were responsible for clinical data acquisition. Chhaya Barapatre performed record analysis, organized figures and tables and ensured ethical standards were met. Arvind Nair conducted the final review, critically revised the manuscript and facilitated the acquisition of funding. All authors reviewed and approved the final version of the manuscript.

## Funding:

None

## Institutional review board statement:

Ethical approval was acquired before initiating the study with IEC Proposal No. 07/GDC/Institutional Ethics Committee/2023.
